# Uneven progress: trends and social inequalities in women’s empowerment domains in Ghana from 2003–2022

**DOI:** 10.3389/fsoc.2026.1811952

**Published:** 2026-06-26

**Authors:** Yula Salifu, Torjim Salifu, Adwoa Asieduwaa Boakye, Mubaric Yakubu, Amidu Alhassan, Joseph Lasong

**Affiliations:** 1 Department of Population, Family and Reproductive Health, School of Public Health, University of Ghana, Accra, Ghana; 2 Department of Nursing, St. Patrick’s Hospital, Offinso, Ghana; 3 Department of Population, Family and Reproductive Health, School of Public Health, University of Technology and Applied Sciences, Navrongo, Ghana; 4 Department of Adult Health, School of Nursing and Midwifery, University of Cape Coast, Cape Coast, Ghana; 5 Department of Population and Reproductive Health, School of Public Health, University for Development Studies, Tamale, Ghana

**Keywords:** decision-making, inequality, social independence, sustainable development goals, violence, women empowerment

## Abstract

**Introduction:**

Despite notable policy efforts, evidence on long-term trends and social inequalities in women’s empowerment in Ghana remains limited. This study examined changes from 2003 to 2022 across three SWPER-derived domains including social independence, decision-making, and attitudes toward violence, focusing on regional, socioeconomic, age, education, and urban–rural disparities.

**Methods:**

Data from the Ghana Demographic and Health Survey waves (2003–2022) was used to estimate the prevalence of high empowerment among currently married women aged 15–49 years. Inequalities were assessed using Difference (D), Ratio (R), Population Attributable Fraction (PAF), Population Attributable Risk (PAR), Absolute Concentration Index (ACI), and Theil Index (TI), with trends disaggregated by region, wealth, education, age, and residence.

**Results:**

Empowerment prevalence increased across all domains, but trajectories diverged. Attitudes toward violence improved substantially (61.6% to 79.2% between 2008 and 2022), accompanied by narrowing regional and wealth inequalities. Decision-making rose to 61.4% in 2014 before declining to 55.6% in 2022, with widening age and wealth disparities thereafter. Social independence increased from 29.5 to 45.5% but remained the most unequal domain: a 45-percentage-point gap persisted between Greater Accra (67.2%) and North-East (22.4%), while adolescent coverage stagnated below 5%. Education was the dominant driver of inequality in social independence (PAF > 115%; PAR ≈ 53 percentage points). Although TI declined across domains, rising ACI for education indicated increasingly concentrated gains among educated women.

**Conclusion:**

While Ghana has achieved normative progress and reduced geographic disparities, substantial socioeconomic and age-related inequalities persist, particularly in social independence, necessitating equity-focused, domain-specific policy responses beyond national averages.

## Introduction

Women’s empowerment, defined as the process by which women gain the ability to make strategic life choices and exercise agency over their lives, is a fundamental human right and a critical driver of sustainable development (Kabeer, 1999; United Nations, 2015). It is enshrined as a central target of Sustainable Development Goal (SDG) 5, recognizing that gender equality and women’s empowerment are pivotal for achieving health, economic, and social progress across all nations (UN Women, 2022). Despite this global commitment, progress remains slow and uneven. Recent evidence indicates that at the midpoint of the 2030 Agenda, SDG 5 is alarmingly off-track, with estimates suggesting it could take centuries to close gaps in legal protection and political representation under current trajectories (UN Women, 2024). This stagnation is fuelled by pervasive structural barriers, including discriminatory social norms, unequal laws, and the disproportionate burden of unpaid care work borne by women (Nigah, 2024).

The empowerment of women is not a monolithic concept but a multidimensional construct encompassing resources, agency, and achievements (Kabeer, 1999). This has informed the development of measurement tools like the Survey-based Women’s emPowERment (SWPER) index, which captures three distinct domains critical for Low- and Middle-Income Countries (LMICs): social independence (reflecting education, media access, and life-course autonomy), decision-making (instrumental agency within the household), and attitudes toward violence (intrinsic agency and rejection of harmful norms) (Ewerling et al., 2017). The SWPER index enables comparable, population-level analysis of these domains over time, providing a nuanced picture beyond composite scores (Shannon, 2018).

Globally and across Sub-Saharan Africa (SSA), evidence reveals persistent and deeply entrenched inequalities in women’s empowerment, with significant implications for health and development (Akinwale, 2023). Systematic reviews affirm that higher levels of women’s empowerment are strongly associated with improved maternal and child health outcomes, including better nutrition, higher healthcare utilization, and lower child mortality (Pratley, 2016; Jones et al., 2019). Conversely, disempowerment is a key social determinant of adverse outcomes, mostly illustrated by the high prevalence of gender-based violence (GBV) (Giammarioli et al., 2023). Evidence, from SSA, further demonstrates the cyclical link between economic disempowerment, restrictive gender norms, and intimate partner violence (Ellsberg et al., 2015; Abramsky et al., 2014; Gibbs et al., 2017; Peterman et al., 2022). However, interventions that combine economic empowerment with gender norm transformation show promise in reducing violence and improving women’s agency (Bandiera et al., 2020; Green et al., 2015; Pettifor et al., 2018). Nonetheless, the distribution of empowerment gains and the effectiveness of interventions are highly uneven, often exacerbating existing socioeconomic and geographic inequalities (Ewerling et al., 2017; World Bank, 2021).

However, Ghana, a lower-middle-income country in West Africa, exemplifies these regional patterns of progress punctuated by persistent inequality (Baker, 2016). National policies, such as the Ghana Domestic Violence Act (2007) and girls’ education initiatives, have contributed to measurable improvements in some empowerment indicators (Ghana Statistical Service, Ghana Health Service, and ICF, 2023). Studies using DHS data confirm that factors like education, wealth, and urban residence are strong determinants of women’s autonomy and decision-making power (Abbas et al., 2021). Furthermore, research in Ghana links women’s empowerment to critical outcomes, from rejecting justification of wife-beating (Dickson et al., 2020) to improving access to healthcare (Amankwah, 2015). However, profound disparities still exist. For example, significant gaps persist in women’s control over economic assets like land (Doss et al., 2015), and subnational analyses reveal a stark north–south divide in empowerment outcomes, mirroring broader patterns of regional inequality (Ghana Statistical Service, 2021).

Despite the existence of numerous evidence, critical research gaps remain that this study seeks to address. First, there is a temporal gap, in that; few studies have systematically analysed long-term, multi-wave trends in women’s empowerment in Ghana across the distinct SWPER domains. Second, an inequality gap exists, in that; while socioeconomic and geographic disparities are acknowledged, there is limited analysis quantifying how these inequalities have evolved over two decades using advanced, policy-relevant metrics like the Absolute Concentration Index and Theil Index. Third, a domain-specific analysis gap persists, in that; aggregated empowerment scores can mask divergent trajectories, as progress in attitudes may not correlate with gains in economic independence or decision-making power.

This study therefore aimed to: (1) examine national-level trends in women’s empowerment across three SWPER domains in Ghana from 2003 to 2022; (2) quantify inequalities by age, wealth, education, residence, and region using multiple summary measures; and (3) assess whether empowerment gains have been equitably distributed or concentrated among advantaged groups. The findings are intended to inform targeted, equity-centred policies and programs, offering evidence to accelerate Ghana’s progress toward SDG 5.

## Methods

### Study design

This study employed a repeated cross-sectional descriptive design to examine temporal trends and social inequalities in women’s empowerment in Ghana between 2003 and 2022. The analysis focused on levels, patterns, and changes in empowerment across population subgroups, without causal inference, regression modelling, or decomposition beyond established inequality summary measures. All indicators and inequality metrics were derived from pre-processed estimates available through the World Health Organization (WHO) Health Equity Assessment Toolkit (HEAT) (Osborne et al., 2025).

### Data source

Data for this study were obtained from the WHO Health Equity Assessment Toolkit (HEAT), built-in database edition, Version 6.0 (World Health Organization, 2024). HEAT is a software platform developed by the WHO to support systematic assessment of health inequalities within and between countries (Hosseinpoor et al., 2016).

The HEAT database draws from the WHO Health Inequality Monitor data repository (2024 update), which compiles harmonised secondary analyses of nationally representative household surveys. For Ghana, the underlying data originate from multiple waves of the Demographic and Health Surveys (DHS) conducted between 2003 and 2022. These surveys were implemented by the Ghana Statistical Service (GSS) in collaboration with the Ghana Health Service (GHS) and ICF International, using standardised questionnaires, sampling procedures, and field protocols (Tuoyire et al., 2024).

Ghana is a lower-middle-income country in West Africa with substantial regional, socioeconomic, and urban–rural disparities in health and social outcomes (Dery, 2019). The Ghana Demographic and Health Surveys (DHS) employ a nationally representative, stratified two-stage cluster sampling design and collect standardised data on women’s sociodemographic characteristics, empowerment, reproductive and maternal health, child health, nutrition, household living conditions, and key social determinants of health (Dadzie et al., 2021).

All analyses were conducted within the WHO HEAT platform. Critically, the HEAT built-in database contains pre-processed inequality estimates rather than raw microdata. These estimates were produced by the International Centre for Equity in Health (ICEH) using standardized methodologies across all survey waves. The authors did not perform original calculations on raw DHS microdata; instead, we extracted pre-computed inequality estimates for Ghana from the HEAT database and synthesized these estimates to examine temporal trends and social inequalities across four survey waves (2003–2022).

### Study population

The study population comprised women aged 15–49 years who were married or in a union, consistent with the denominator definition of the Survey-based Women’s Empowerment (SWPER) index. Data were drawn from repeated cross-sectional DHS rounds spanning 2003, 2008, 2014, and 2022, enabling assessment of population-level trends and inequalities over time rather than individual trajectories.

### Women’s empowerment indicators

Women’s empowerment was assessed using the Survey-based Women’s Empowerment (SWPER) index, a validated composite measure developed for low- and middle-income countries and derived from DHS data (Ewerling et al., 2017). The SWPER index comprises three conceptually distinct but complementary domains:

1 Social independence domain

Captures structural and life-course preconditions that enable women to pursue their goals, including educational attainment, access to information, age at first marriage and first birth, and spousal age and education differentials. Women with a domain score >0.293 were classified as having high social independence empowerment.

2 Decision-making domain

Reflects women’s participation in key household decisions, serving as a proxy for instrumental agency. Women with a domain score >0.600 were classified as having high decision-making empowerment.

3 Attitude to violence domain

Measures women’s rejection of norms justifying intimate partner violence, reflecting intrinsic agency and internalised gender norms. Women with a domain score >0.400 were classified as having high empowerment in attitudes toward violence.

Following the validated SWPER methodology (Ewerling et al., 2017, 2020), women with domain scores exceeding the original validation thresholds were classified as having high empowerment: >0.293 for social independence, >0.600 for decision-making, and >0.400 for attitudes toward violence. These cutoffs, derived from an Africa-wide validation study, represent the upper range of standardized scores and have been widely used in comparative research across Sub-Saharan Africa, including Ghana. While no country-specific recalibration has been formally established for Ghana, the SWPER has demonstrated acceptable cross-cultural validity in West African settings.

For each domain, outcomes were expressed as the percentage (%) of women classified as having high empowerment, scaled from 0 to 100, with higher values indicating more favourable empowerment outcomes.

For all prevalence estimates and inequality measures, the HEAT database provides 95% confidence intervals (CIs) derived from the underlying DHS sampling design. These confidence intervals were used to assess the precision and robustness of estimates across subgroups and over time.

Temporal changes and subgroup differences were interpreted cautiously, with attention to the overlap of confidence intervals, particularly for smaller subpopulations such as adolescents and women with higher education. No formal hypothesis testing was performed, and no causal interpretation was implied.

### Equity stratifiers and disaggregation

Empowerment indicators were disaggregated using equity stratifiers recommended by the WHO for health inequality monitoring:

- Age: 15–19 years (adolescents) and 20–49 years
- Economic status: Wealth quintiles (poorest to richest), derived from a household asset-based wealth index
- Educational attainment: No education, primary education, secondary education or higher
- Place of residence: Urban and rural
- Subnational region: Administrative regions of Ghana, based on DHS regional boundaries applicable at each survey wave.

For ordered dimensions such as wealth and education, subgroup ordering was preserved to allow assessment of gradients, rather than binary comparisons alone.

### Statistical analysis

All analyses were conducted within the WHO HEAT platform, which applies appropriate survey weights and accounts for the complex sampling design of the DHS.

For each empowerment domain, national prevalence estimates and subgroup-specific prevalence estimates were generated for all available survey years between 2003 and 2022. Temporal trends were assessed descriptively by comparing point estimates and their associated uncertainty across survey waves.

### Summary measures of inequality

To quantify social and geographic inequalities, both simple and complex inequality measures were employed, following WHO recommendations:

- Difference (D): Absolute difference in prevalence between the most advantaged and most disadvantaged subgroups
- Ratio (R): Relative comparison of prevalence between the most advantaged and disadvantaged subgroups
- Population Attributable Risk (PAR): Absolute potential improvement in national prevalence if all subgroups achieved the level of the most advantaged group
- Population Attributable Fraction (PAF): Relative improvement in national prevalence under the same equity scenario
- Absolute Concentration Index (ACI): Weighted measure summarising inequality across ordered socioeconomic groups, with positive values indicating concentration among advantaged groups. ACI values range from −100 to +100 (theoretically). For binary outcomes expressed as percentages, ACI values above 10 suggest substantial inequality, values between 5 and 10 indicate moderate inequality, and values below 5 indicate relatively low inequality. Positive ACI values indicate pro-rich or pro-educated concentration
- Theil Index (TI): A measure of between-region inequality, sensitive to disparities at the extremes of the distribution. The Theil Index theoretically ranges from 0 (perfect equality, all regions have identical prevalence) to infinity (maximum inequality). Higher values indicate greater between-region variation. The index is unitless, and comparisons are meaningful only within the same outcome and population over time. A declining Theil Index indicates convergence in regional outcomes.

Difference and PAR are absolute measures, while Ratio and PAF are relative measures. The ACI and TI are complex measures that incorporate information across all subgroups rather than only extremes. The use of both absolute and relative measures was considered essential to avoid misleading conclusions arising from reliance on a single metric.

### Ethical considerations

Ethical approval was not required for this study, as it relied exclusively on publicly available, anonymised secondary data. All DHS surveys were approved by the relevant national ethical review bodies in Ghana and by ICF International, and were conducted in accordance with international ethical standards, with informed consent obtained from all participants at the time of data collection.

The Strengthening the Reporting of Observational Studies in Epidemiology (STROBE) guidelines were used in reporting data in this study (Von elm et al., 2007).

## Results

### Inequalities in social-independence domain among women with high empowerment by region

**Figure 1:**
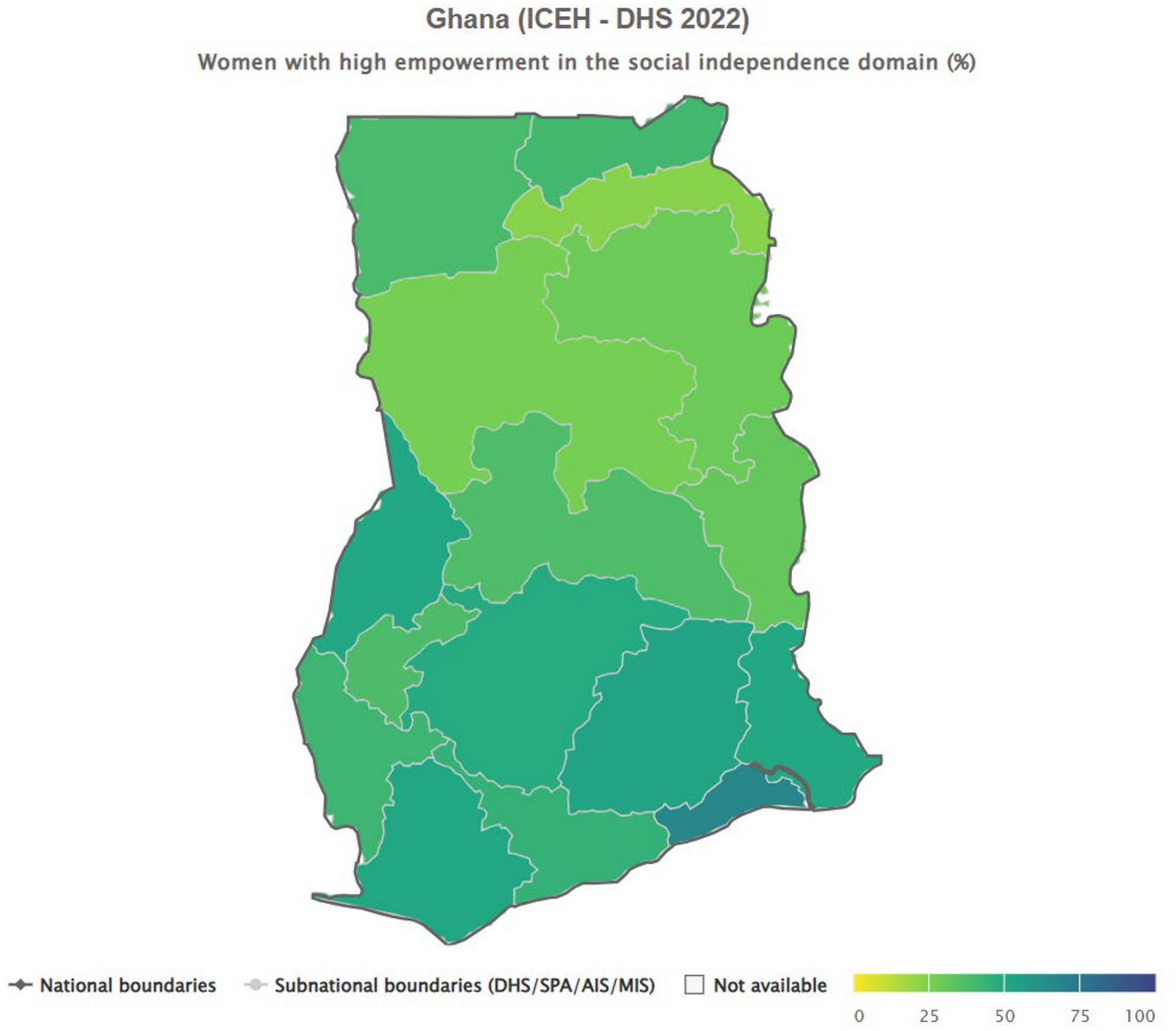
Inequalities in social independence domain among women with high empowerment by region, 2022. Source: https://www.who.int/data/inequality-onitor/assessment_toolkit (World Health Organization, 2024).

Figure 1 highlights the inequality in women empowerment across the social independence domain. In 2022, the prevalence of women with high empowerment in the social independence domain varied across subnational regions in Ghana, indicating substantial geographic inequality. At the national level, 45.5% of women were classified as having high social independence empowerment. The highest prevalence was observed in Greater Accra, where 67.2% of women were highly empowered (95% CI: 61.7–72.3). Other regions with comparatively high levels included the Eastern Region (52.3%; 95% CI: 46.5–58.1), Western Region (50.4%; 95% CI: 44.2–56.7), Bono (50.0%; 95% CI: 42.8–57.1), Volta (49.6%; 95% CI: 43.9–55.3), and Ashanti (48.5%; 95% CI: 43.0–54.0). Nonetheless, moderate levels of empowerment were observed in Central Region (44.2%; 95% CI: 36.9–51.8), Western North (41.8%; 95% CI: 35.4–48.5), Upper East (40.4%; 95% CI: 35.9–45.0), Upper West (38.6%; 95% CI: 32.4–45.2), Ahafo (37.5%; 95% CI: 32.6–42.7), and Bono East (37.0%; 95% CI: 32.4–41.9). Also, the lowest prevalence of high social independence empowerment was recorded in the northern belt of the country. North-East Region had the lowest estimate at 22.4% (95% CI: 18.6–26.6), followed by Savannah (26.2%; 95% CI: 21.1–32.1), Northern Region (28.8%; 95% CI: 23.6–34.7), and Oti Region (30.9%; 95% CI: 26.7–35.5). However, the absolute difference between the highest- and lowest-performing regions was substantial, with a gap of approximately 45 percentage points between Greater Accra and North-East (Figure 1).

### Inequalities in decision-making domain among women with high empowerment by region

**Figure 2:**
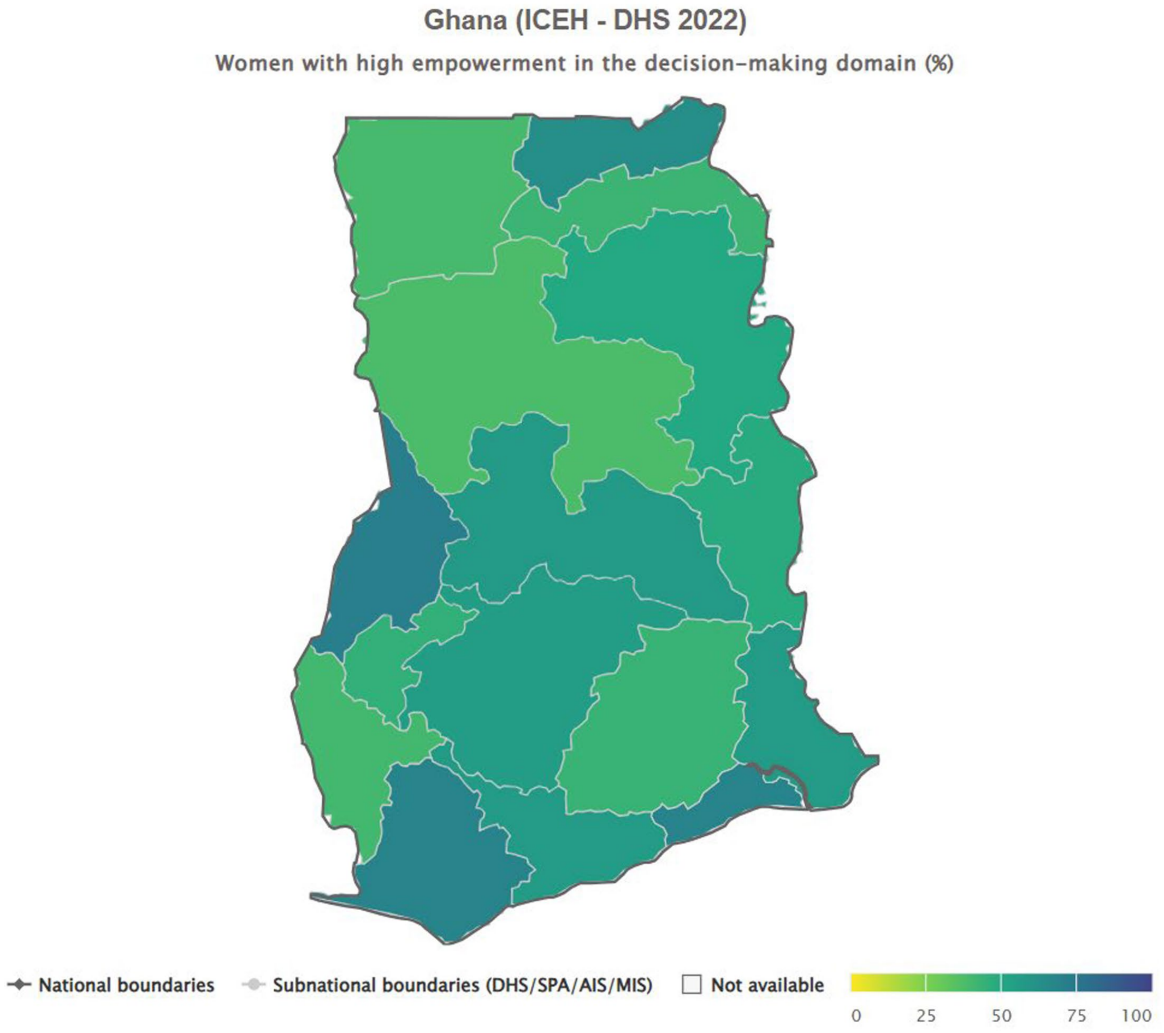
Inequalities in decision-making domain among women with high empowerment by region, 2022. Source: https://www.who.int/data/inequality-onitor/assessment_toolkit (World Health Organization, 2024).

Figure 2 highlights the inequality in women empowerment across the decision-making domain. In 2022, the proportion of women with high empowerment in the decision-making domain exhibited considerable variation across subnational regions in Ghana. The national average prevalence of high decision-making empowerment was 55.6%. The highest regional prevalences were observed in Bono Region, where 71.5% of women were classified as highly empowered (95% CI: 64.4–77.6), Greater Accra (68.8%; 95% CI: 63.7–73.4), and Western Region (68.7%; 95% CI: 60.9–75.6). High levels were also recorded in Upper East (63.7%; 95% CI: 55.8–71.0). However, several regions recorded prevalence estimates close to the national average. These included Ashanti (55.7%; 95% CI: 50.1–61.2), Central (56.9%; 95% CI: 51.2–62.3), Volta (56.9%; 95% CI: 50.7–62.8), and Bono East (57.1%; 95% CI: 47.8–66.0). Nonetheless, lower prevalences of high decision-making empowerment were observed in several regions. Eastern Region recorded 43.4% (95% CI: 37.8–49.1), while North-East had 42.4% (95% CI: 35.6–49.5). Similarly, Western North (40.2%; 95% CI: 33.2–47.7), Upper West (39.2%; 95% CI: 30.0–49.2), Savannah (38.0%; 95% CI: 31.0–45.5), and Ahafo (45.2%; 95% CI: 35.0–55.8) all recorded point estimates below the national average, with confidence intervals largely falling beneath or overlapping the national estimate. Overall, the absolute regional gap between the highest- and lowest-performing regions was substantial. The difference between Bono Region (71.5%) and Savannah Region (38.0%) was approximately 33 percentage points (Figure 2).

### Inequalities in attitude to violence domain among women with high empowerment by region

**Figure 3:**
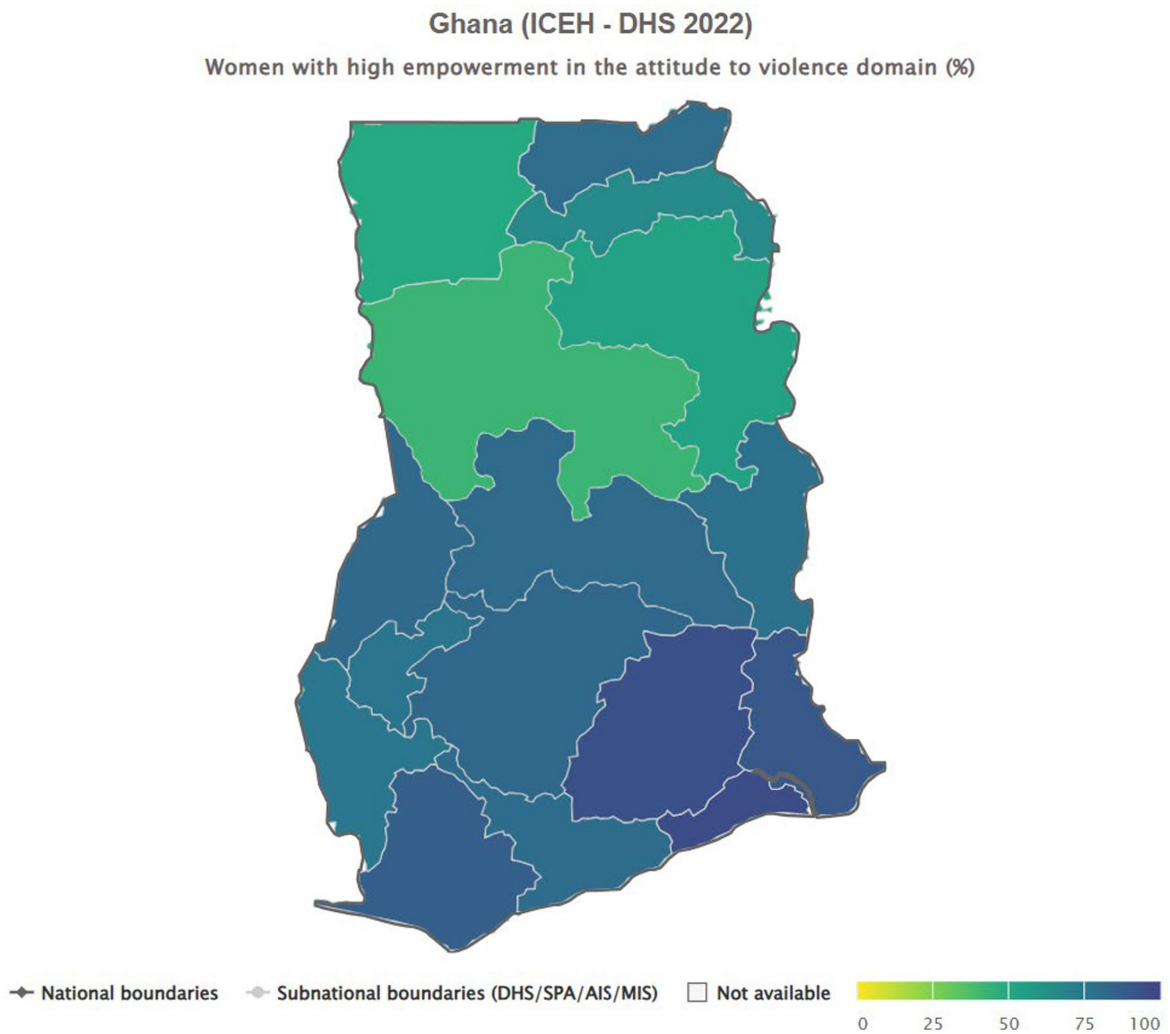
Inequalities in attitude to violence domain among women with high empowerment by region, 2022. Source: https://www.who.int/data/inequality-onitor/assessment_toolkit (World Health Organization, 2024).

Figure 3 highlights the inequality in women empowerment across the attitude towards violence domain. In 2022, the prevalence of women with high empowerment in the attitude to violence domain varied substantially across subnational regions in Ghana. The national average proportion of women classified as highly empowered in this domain was 79.2%. The highest prevalences were observed in Greater Accra, where 95.5% of women reported high empowerment (95% CI: 92.4–97.4), followed closely by the Eastern Region (93.6%; 95% CI: 90.9–95.5) and Volta Region (89.8%; 95% CI: 84.2–93.5). High levels were also recorded in Western Region (86.5%; 95% CI: 81.8–90.2) and Ashanti Region (82.9%; 95% CI: 77.8–87.1). Also, several regions recorded levels of empowerment near the national average. These included Upper East (80.0%; 95% CI: 71.4–86.4), Central Region (80.3%; 95% CI: 76.3–83.7), Oti Region (78.4%; 95% CI: 72.7–83.3), Bono Region (81.6%; 95% CI: 74.0–87.3), and Bono East (81.2%; 95% CI: 69.2–89.3). Nonetheless, lower prevalences of high empowerment in attitudes toward violence were observed in several regions. Ahafo Region recorded 76.3% (95% CI: 71.3–80.7), while Western North had 75.7% (95% CI: 69.7–80.9). Markedly lower estimates were observed in the northern belt, including North-East (65.4%; 95% CI: 58.2–72.0), Northern Region (52.6%; 95% CI: 48.1–57.1), Upper West (49.1%; 95% CI: 42.6–55.6), and Savannah Region, which recorded the lowest prevalence at 42.6% (95% CI: 31.4–54.6). In these regions, confidence intervals were substantially below the national average. Overall, the absolute regional gap between the highest- and lowest-performing regions was pronounced. The difference between Greater Accra (95.5%) and Savannah Region (42.6%) was approximately 53 percentage points (Figure 3).

### Inequalities in women empowerment domains by age, economic status, sub-region, education and place of residence

#### Attitude to violence domain

**Figure 4:**
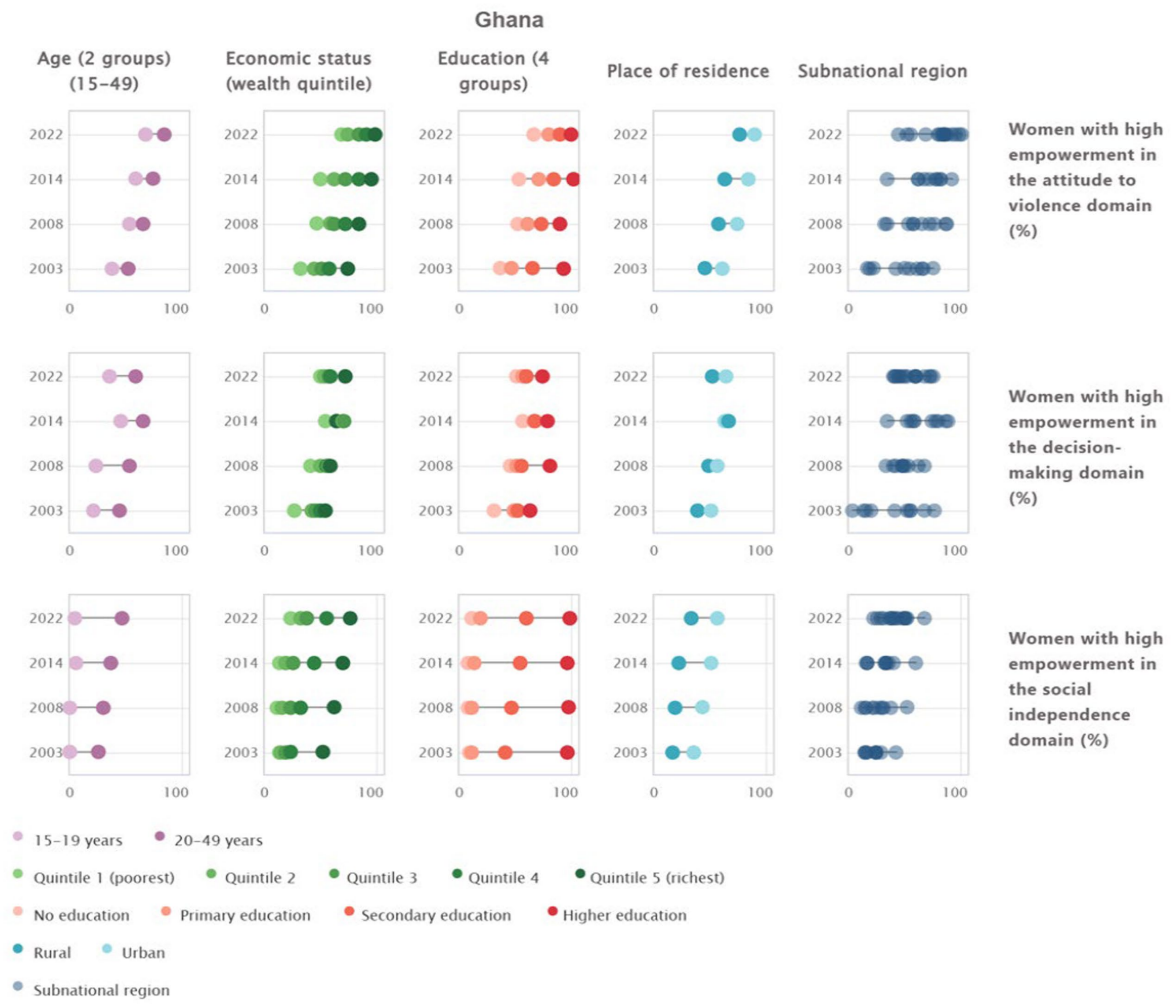
Inequalities in women empowerment domains by age, economic status, sub-region, education and place of residence.

At the national level, the prevalence of women with high empowerment in the attitude to violence domain increased steadily over time, rising from 61.6% (95% CI: 59.4–64.4) in 2008, to 70.0% (95% CI: 67.2–73.2) in 2014, and further to 79.2% (95% CI: 78.0–81.0) in 2022. Across all survey waves, women aged 20–49 years consistently exhibited higher empowerment than adolescents aged 15–19 years. In 2008, prevalence among adolescents was 51.0% (95% CI: 38.9–63.1) compared with 61.9% (95% CI: 59.4–64.4) among adults. By 2014, this increased to 56.1% (95% CI: 44.3–67.3) for adolescents and 70.3% (95% CI: 67.2–73.2) for adults. In 2022, the gap persisted, with adolescents at 63.8% (95% CI: 56.3–70.8) and adults at 79.5% (95% CI: 78.0–81.0). However, a strong and persistent wealth gradient was evident across all timelines. In 2008, prevalence ranged from 44.6% (95% CI: 39.8–49.4) in the poorest quintile to 79.5% (95% CI: 74.5–83.7) in the richest. This gradient widened in absolute terms by 2022, with estimates increasing from 65.1% (95% CI: 61.1–68.9) in the poorest quintile to 92.8% (95% CI: 90.7–94.4) in the richest quintile. Also, educational inequalities were pronounced and persistent. In 2008, women with no education had a prevalence of 49.4% (95% CI: 45.0–53.8) compared with 84.8% (95% CI: 73.7–91.7) among those with higher education. By 2022, this gap remained substantial, ranging from 63.1% (95% CI: 60.1–66.0) among women with no education to 94.6% (95% CI: 92.0–96.4) among those with higher education. Urban women consistently exhibited higher empowerment than rural women across all years. The urban–rural inequality gap widened from 14.6 percentage points in 2003 to a peak of 19.3 points in 2014, before narrowing to 12.7 points in 2022 for urban women compared to rural women (Figure 4).

#### Decision-making domain

Nationally, empowerment in the decision-making domain followed a non-linear temporal pattern. Prevalence increased from 49.7% (95% CI: 47.8–53.2) in 2008 to 61.4% (95% CI: 59.3–64.2) in 2014, before declining slightly to 55.6% (95% CI: 54.1–58.1) in 2022. However, age disparities were substantial and persistent. In 2008, only 22.0% (95% CI: 13.9–33.2) of adolescents had high decision-making empowerment, compared with 50.5% (95% CI: 47.8–53.2) of adult women. Although adolescent empowerment improved to 42.8% (95% CI: 31.5–54.9) in 2014, it declined again to 33.4% (95% CI: 25.9–41.8) in 2022, widening the age gap once more. Also, in 2008, prevalence ranged from 38.9% (95% CI: 34.0–44.1) in the poorest quintile to 56.0% (95% CI: 50.4–61.4) in the richest quintile. By 2014, the gradient narrowed, but by 2022 it widened again, with estimates increasing from 47.5% (95% CI: 43.0–52.0) in the poorest quintile to 67.9% (95% CI: 64.1–71.5) in the richest quintile. Again, educational gradients were evident across all timelines. In 2022, women with no education had a prevalence of 48.3% (95% CI: 44.2–52.4), compared with 70.3% (95% CI: 65.7–74.5) among women with higher education. Urban–rural differences persisted but were less pronounced than for education or wealth. Urban prevalence increased from 48.3% (2003) to 61.1% (2022). Rural prevalence increased from 36.7% (2003) to 62.7% in 2014 but then declined to 49.7% in 2022 (Figure 4).

#### Social independence domain

The social independence domain consistently recorded the lowest empowerment levels, but showed steady improvement over time, increasing from 29.5% (95% CI: 28.0–32.7) in 2008, to 36.5% (95% CI: 34.7–39.6) in 2014, and to 45.5% (95% CI: 44.6–48.4) in 2022. Adolescents remained markedly disadvantaged across all years. In 2008, only 0.5% (95% CI: 0.1–3.8) of adolescents had high social independence empowerment, compared with 30.3% (95% CI: 28.0–32.7) among adult women. Although adolescent empowerment increased slightly to 5.9% (95% CI: 1.5–20.9) in 2014 and 4.5% (95% CI: 2.4–8.4) in 2022, absolute levels remained extremely low. Additionally, economic inequalities were pronounced and widened over time. In 2008, prevalence ranged from 11.8% (95% CI: 9.2–15.0) in the poorest quintile to 31.9% (95% CI: 27.6–36.6) in the richest. By 2022, this disparity had expanded substantially, from 23.6% (95% CI: 21.3–25.9) in the poorest quintile to 75.6% (95% CI: 72.0–78.9) in the richest quintile. Also, educational gradients were extreme. In 2022, only 11.3% (95% CI: 9.8–13.1) of women with no education had high social independence empowerment, compared with 98.4% (95% CI: 95.7–99.4) among women with higher education. Urban women consistently had substantially higher empowerment than rural women across all survey waves. Urban empowerment more than doubled from 35.2% in 2003 to 56.4% in 2022. Rural empowerment also increased but from a much lower base, from 17.1 to 33.9% (i.e., the urban–rural gap widened over time, from 18.1 points in 2003 to a peak of 27.8 points in 2014, before narrowing slightly to 22.5 points in 2022) (Figure 4).

### Trends and social inequalities in empowerment domains among women with high empowerment in Ghana, 2003–2022

#### Difference (D)

The Difference (D) measures the absolute inequality between the most and least advantaged subgroups (percentage points).

#### Attitude to violence

**Figure 5:**
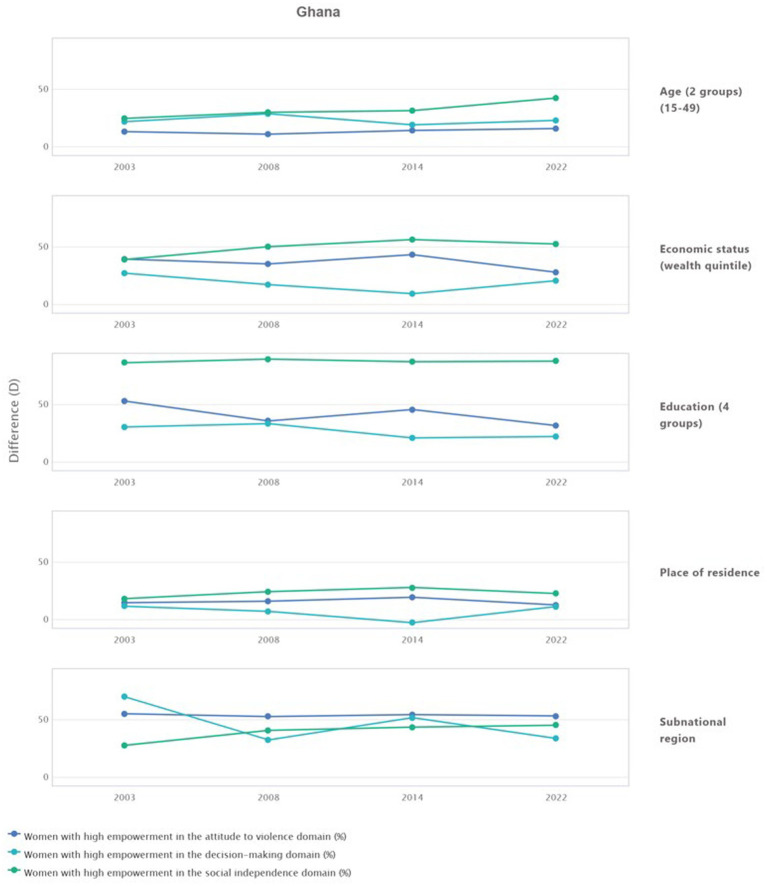
Population coverage for inequality analysis by dimension (Difference).

The widest gaps were consistently observed across subnational regions, with differences exceeding 50 percentage points in all years (e.g., 54.8 in 2003, 52.9 in 2022). Economic status and education followed, with differences of 27.7–42.9 points. Wealth-based inequality in this domain showed a clear decreasing trend, from D = 39.1 in 2003 to 27.7 in 2022. The urban–rural gap also narrowed from 14.6 to 12.7 points (Figure 5).

#### Decision-making

Subnational region again showed the largest absolute gaps, particularly in 2003 (D = 69.5). Age was also a major source of inequality, with differences exceeding 20 points (e.g., 28.5 in 2008, 22.7 in 2022). The urban–rural difference was highly variable, peaking at 11.5 points (2003), disappearing in 2014 (D = -2.6, favouring rural), and re-emerging at 11.3 points in 2022 (Figure 5).

#### Social independence

Education generated by far the largest absolute gaps, reaching 87.0 points in 2022. Economic status and age were also major sources, with differences of 52.1 and 42.0 points, respectively, in 2022. Also, educational inequality remained extremely high but showed a slight decrease from its peak of 88.8 points in 2008. The age gap widened dramatically, from 24.5 points in 2003 to 42.0 points in 2022 (Figure 5).

#### Ratio (R)

The Ratio (R) measures the relative inequality between the most and least advantaged subgroups.

#### Attitude to violence

**Figure 6:**
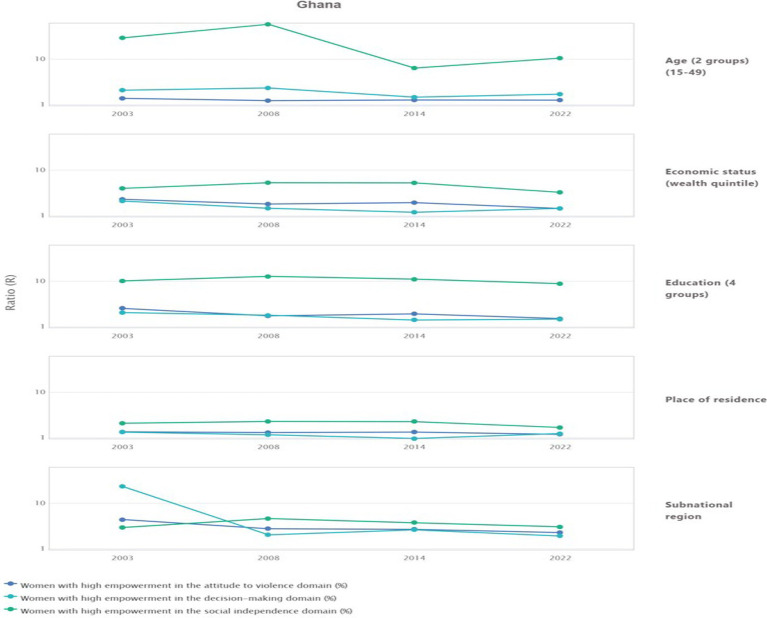
Population coverage for inequality analysis by dimension (Ratio).

Subnational regions showed extreme relative inequality in early years (R = 4.33 in 2003), which decreased to 2.24 by 2022. Economic status ratios were stable at moderate levels (1.43–1.91 across years). Relative inequality by region, education, and wealth all showed a general declining trend, indicating a reduction in the proportionate gap between extremes (Figure 6).

#### Decision-making

Age disparities produced very high relative inequality, especially for adolescents, with ratios of 2.29 (2008) and 1.68 (2022). Subnational region ratios were also high, around 2.00 in most years. The urban–rural ratio was exactly 1.0 (no inequality) in 2014, reflecting the unique crossover where rural women had slightly higher empowerment (Figure 6).

#### Social independence

This domain exhibited the most severe relative inequalities. The age ratio was high in 2008 (R = 56.45) due to near-zero adolescent prevalence, moderating to 10.31 in 2022. The education ratio remained high, at 8.67 in 2022, meaning women with higher education were nearly nine times more likely to be empowered than those with no education. Despite some fluctuations, relative inequalities by education, wealth, and residence remained persistently high over the two decades, with no consistent downward trend (Figure 6).

#### Population attributable fraction (PAF)

The PAF estimates the proportional reduction in the national average that is attributable to inequality (i.e., if all groups had the rate of the best-off group, the average would be X% higher).

PAF values were consistently highest for the Social Independence domain and lowest for Attitude to Violence, reflecting the former’s highest gradients. For social Independence, education was the dominant attributable factor, with PAFs above 115% in all years (e.g., 115.98% in 2022).

The Population Attributable Fraction (PAF) estimates the proportional reduction in the national average that is theoretically achievable if all subgroups attained the empowerment level of the most advantaged group. In this analysis, PAF values exceeding 100% occur when the reference group (e.g., women with higher education) has empowerment prevalence approaching 100% and the national average is substantially lower. This does not indicate a mathematical error but rather that the absolute gap between the reference group and the national average exceeds the national average itself. Such values should be interpreted as “theoretical maximum improvements” rather than literal statements about doubling. For example, a PAF of 115.98% indicates that eliminating the educational gap in social independence would theoretically increase the national average by approximately 116% of its current value, equivalent to more than doubling the prevalence.

**Figure 7:**
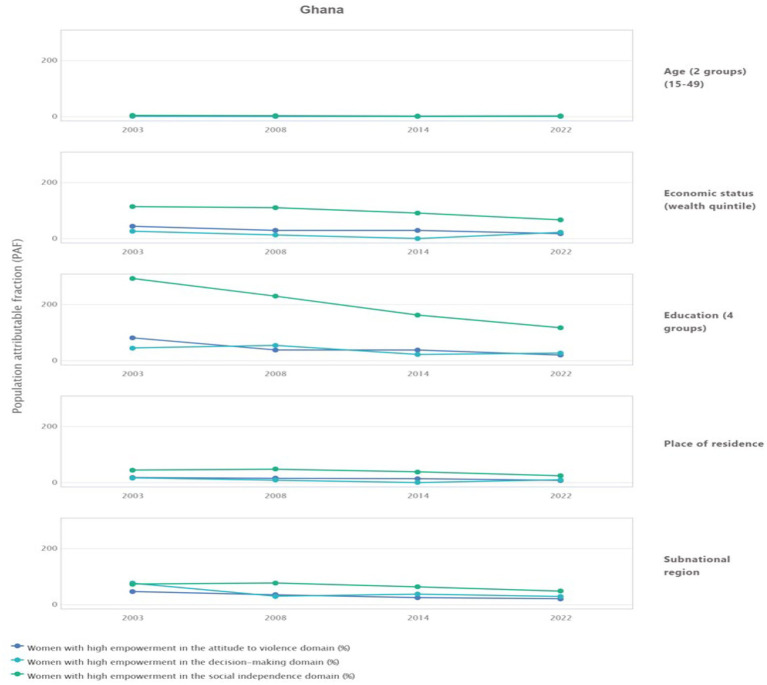
Population coverage for inequality analysis by dimension (Population Attributable Fraction).

Economic status was also a major driver for this domain (PAF ~ 66% in 2022) (Figure 7). There was a notable decrease in PAF for most dimensions across all domains from 2003 to 2022. For example: education PAF for Attitude to Violence fell from 80.21% to 19.50%, while wealth PAF for Social Independence fell from 113.06% to 66.05%. This indicates that while absolute differences remain, the proportion of the total burden of low empowerment explained by these inequalities has reduced over time (Figure 7).

#### Population attributable risk (PAR)

The PAR estimates the absolute increase in the national average (percentage points) if all groups had the rate of the best-off group.

**Figure 8:**
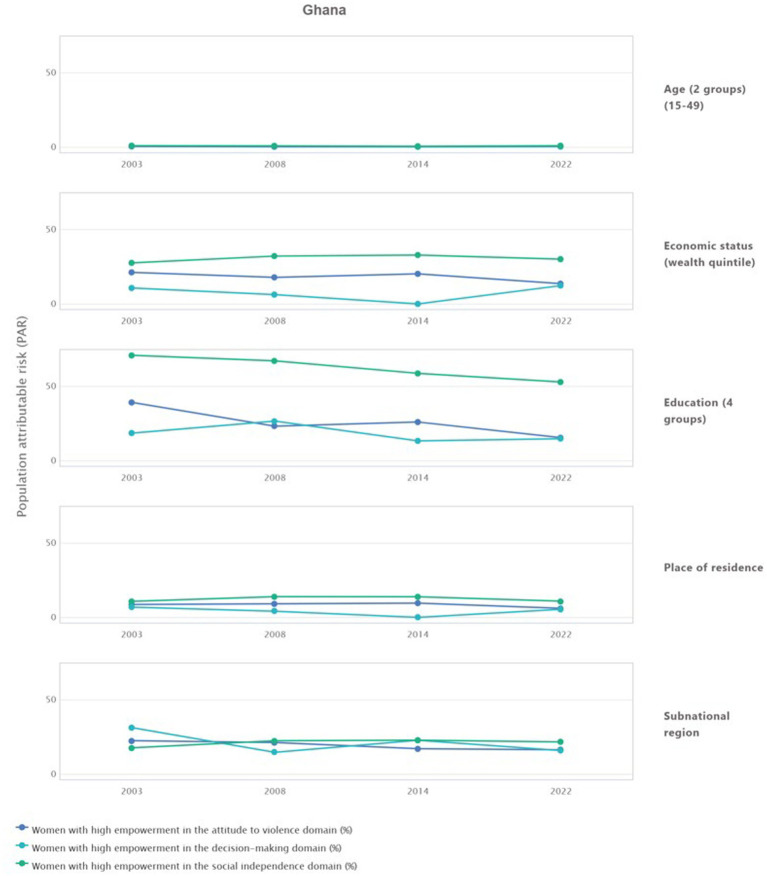
Population coverage for inequality analysis by dimension (Population Attributable Risk).

PAR trends generally mirrored those of the Difference (D) but are weighted by subgroup population sizes. The largest potential gains from eliminating inequality were consistently in the Social Independence domain, particularly from education (PAR of 52.82 points in 2022) and wealth (PAR of 30.08 points in 2022). PAR values for education and wealth in the Attitude to Violence and Decision-Making domains showed a general decline (e.g., Education PAR for Attitude to Violence fell from 39.14 points in 2003 to 15.44 in 2022), signalling a reduction in the population-level impact of these inequalities. Also, PAR for Social Independence remained high (Figure 8).

#### Absolute concentration index (ACI)

The ACI measures socioeconomic inequality related to economic status or education, considering the distribution across all ordered groups (not just extremes).

ACI values range from −100 to +100, with positive values indicating concentration among more advantaged groups (higher wealth or education). Values above 10 suggest substantial inequality, values between 5 and 10 indicate moderate inequality, and values below 5 indicate relatively low inequality.

**Figure 9:**
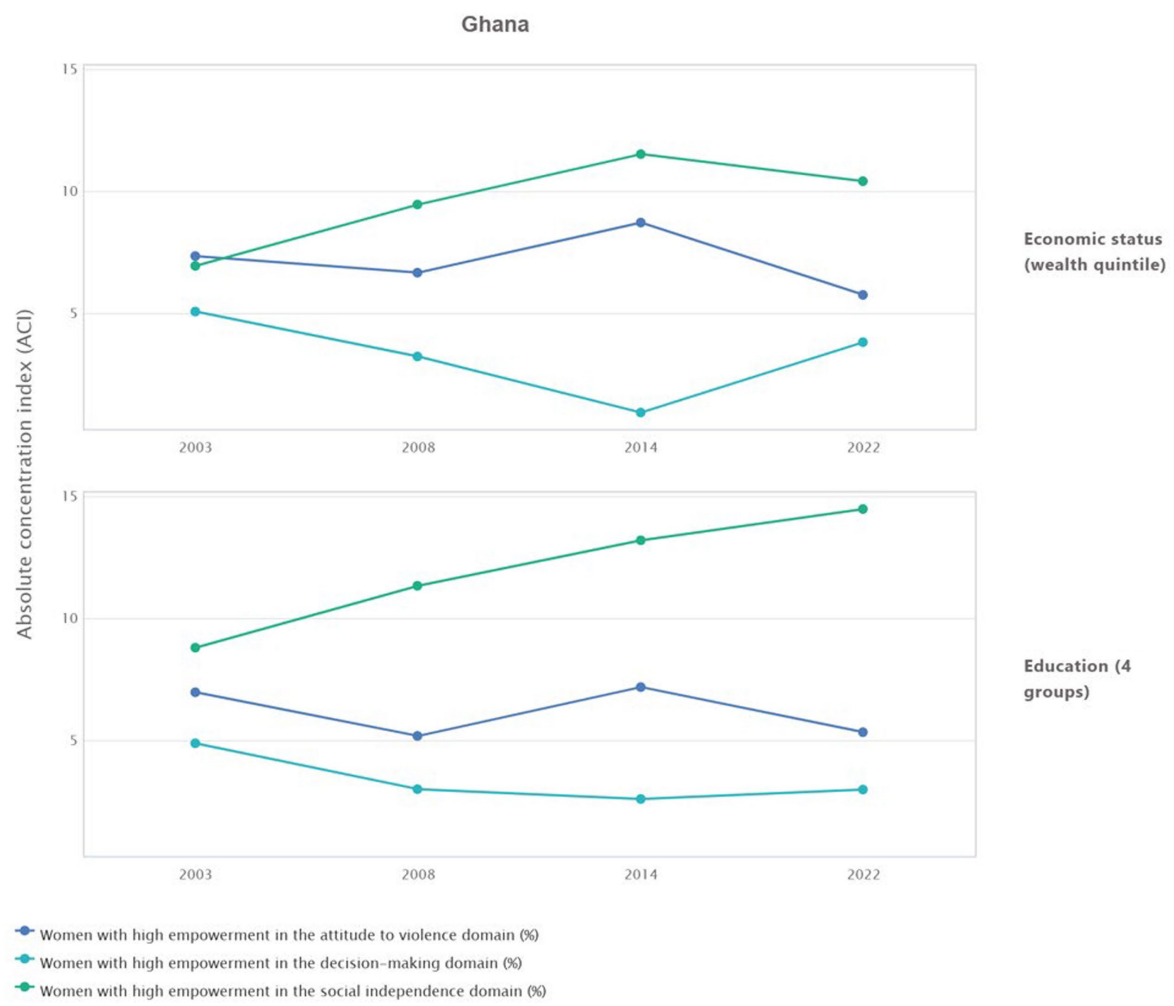
Population coverage for inequality analysis by dimension (Absolute Concentration Index).

ACI values were overwhelmingly positive and largest for the Social Independence domain, confirming strong pro-rich and pro-educated gradients. Under Social Independence domain in 2022, education ACI was 14.50 and Wealth ACI was 10.42, indicating intense concentration of empowerment among the highly educated and wealthy. Also, under the Attitude to Violence in 2022, moderate concentration (ACI: 5.75 for wealth, 5.33 for education). Moreover, under Decision-Making domain in 2022, lowest concentration (ACI: 3.81 for wealth, 2.98 for education) were recorded. Nonetheless, for Attitude to Violence, the wealth ACI decreased from 7.35 (2003) to 5.75 (2022). For Social Independence, the education ACI increased from 8.80 (2003) to 14.50 (2022), suggesting a worsening in the distribution of social independence, with gains increasingly concentrated among the highly educated (Figure 9).

#### Theil index (TI)

The Theil Index (TI) measures overall inequality between subnational regions, ranging from 0 (perfect equality) to infinity (maximum inequality). Higher values indicate greater between-region variation. A declining TI indicates convergence in regional outcomes.

**Figure 10:**
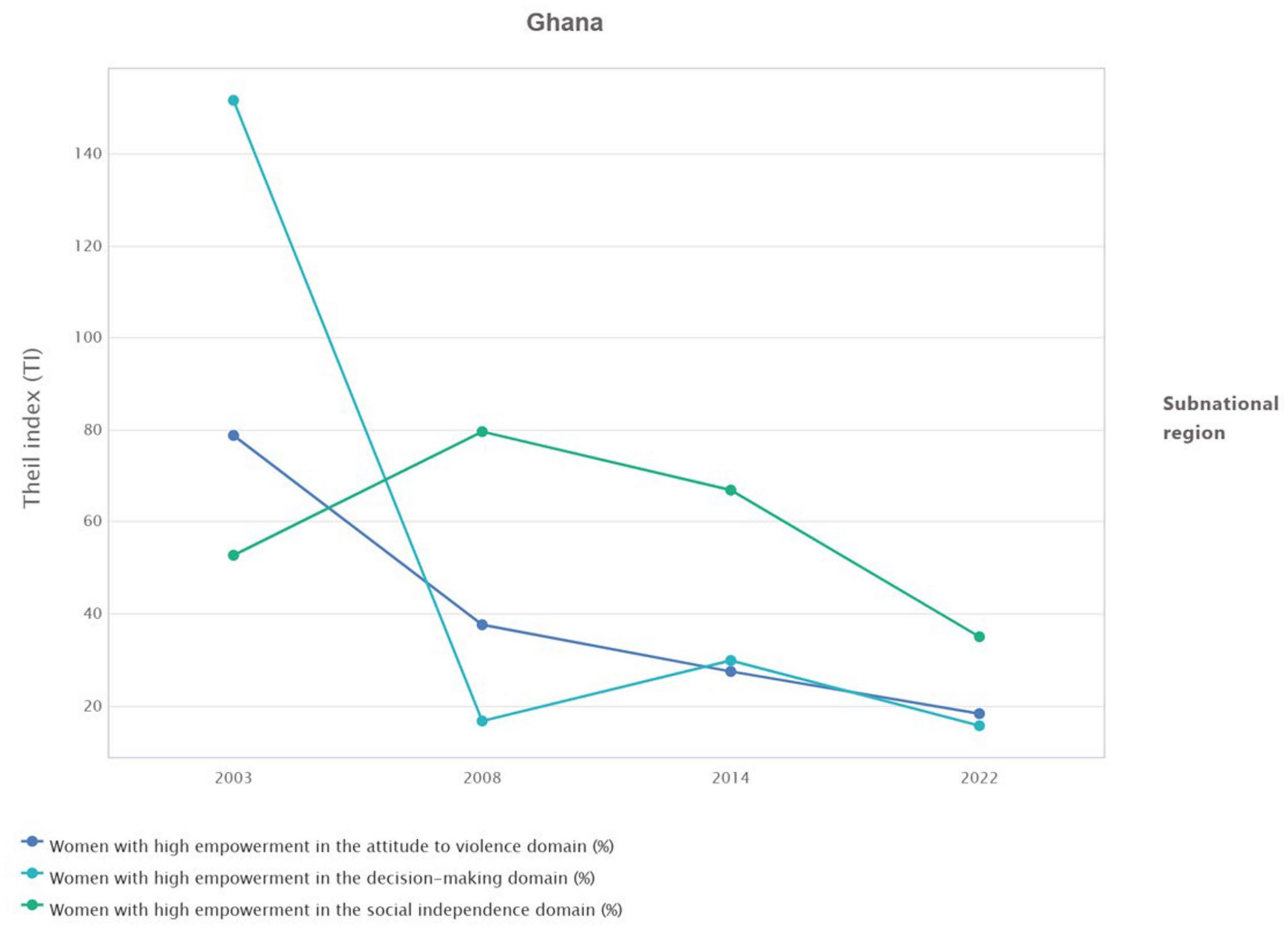
Population coverage for inequality analysis by dimension (Theil Index).

The most consistent trend across all data is the uniform decline in the Theil Index for all three empowerment domains between 2003 and 2022, in that: for attitude to Violence, TI fell from 78.67 to 18.21. For Decision-Making, TI fell from 151.49 to 15.58 and for Social Independence, TI fell from 52.62 to 34.92. This indicates a substantial reduction in geographic inequality (between-region variation) over the two decades. The distribution of women’s empowerment across Ghana’s regions has become significantly more equitable, even as socioeconomic inequalities within regions persist (Figure 10).

## Discussion

### Summary of findings

This study presents a comprehensive, two-decade analysis (2003–2022) of women’s empowerment inequalities in Ghana, examining three SWPER-derived domains: social independence, decision-making, and attitudes toward violence. Nationally, empowerment has improved across all domains, yet these gains have been unevenly distributed. Geographic disparities have narrowed substantially over time, suggesting effective diffusion of national policies and infrastructural improvements. However, socioeconomic inequalities, particularly related to education, wealth, and age, persist and, in some domains, have intensified. Adolescents, less-educated women, poorer households, and residents of northern and rural regions remain consistently disadvantaged.

The findings reveal distinct trajectories across domains. Attitudes toward violence show broad progress and declining inequalities, decision-making displays non-linear trends with intermittent reversals, and social independence remains the most inequitable domain with persistent pro-rich and pro-educated concentration. These patterns highlight the possible mechanisms shaping empowerment in Ghana and reveals critical areas for targeted intervention.

### Trends and inequality measures in the factors associated with women empowerment

#### Social independence

Social independence, which encompasses mobility, asset ownership, access to information, and time allocation, remains stark to persistent inequality. Nationally, 45.5% of women in 2022 were classified as highly empowered in this domain, yet regional variation was extreme: Greater Accra (67.2%) versus North-East (22.4%), reflecting a 45-percentage-point gap. Adolescents fared worst, with only 4.5% highly empowered, highlighting possible life-cycle vulnerabilities.

Also, education and wealth were key determinants. Women with higher education were nearly nine times more likely to be empowered than women with no formal education, and the richest households enjoyed over threefold higher empowerment than the poorest. Urban–rural differences persist, albeit with modest improvements in rural areas over time. These disparities echo patterns observed in Sub-Saharan Africa, where structural inequalities, limited economic opportunities, and restrictive sociocultural norms constrain women’s autonomy, particularly in northern regions (Upadhyay et al., 2014; Ampofo, 2017; Ghana Statistical Service, 2021).

The increasing Absolute Concentration Index (ACI) for education in social independence (from 8.8 to 14.5) indicates that gains are disproportionately captured by the most educated women, leaving poorer and less-educated women increasingly marginalized. This finding contrasts with some South Asian contexts, where targeted microfinance and large-scale employment programs have demonstrated greater penetration and more equitable distribution of economic agency among poorer women (Nayar et al., 2011; Murshid, 2018). Social independence requires material resources and freedom from time poverty, which remain inaccessible to the most disadvantaged, emphasizing the need for targeted economic and structural interventions. Additionally, research should explore mechanisms underpinning persistent adolescent and regional disadvantage, including qualitative investigations of mobility restrictions, asset control, and cultural norms. Public policies should also prioritize structural interventions, such as education scholarships, skills training, land rights, financial inclusion programs, and youth empowerment initiatives, particularly in northern and rural areas. It would be prudent to further integrate empowerment modules into health and social services, focusing on building adolescents’ mobility, decision-making, and economic skills.

#### Decision-making

Decision-making empowerment, which reflects a woman’s ability to influence household choices, showed a non-linear trajectory. National prevalence peaked at 61.4% in 2014 but declined to 55.6% in 2022, with notable declines among adolescents (33.4% in 2022). Regional variation ranged from 71.5% in Bono to 38.0% in Savannah, while urban–rural differences were moderate. Education and wealth gradients persisted, though relative inequalities were less extreme than in social independence.

This pattern aligns with broader West African evidence, where household decision-making is strongly shaped by age and economic capacity, yet remains limited for younger and poorer women (Gnoumou Thiombiano, 2014; Amoak et al., 2025; Kingsley, 2023; Boateng et al., 2014). The temporary urban–rural parity observed in 2014 may reflect the impact of short-term rural livelihood programs, the effects of which were possibly not sustained. The volatility of this domain may highlight its sensitivity to household dynamics, social norms, and program effectiveness (Kabeer, 2005; Jones et al., 2019).

The findings contrast with steadier progress observed in some Southeast Asian contexts (Nayar et al., 2011; Murshid, 2018), highlighting the vulnerabilities embedded in West African patriarchal family structures and the precariousness of gains in women’s bargaining power (Boateng et al., 2014).

Future research should investigate drivers of adolescent decline and temporal volatility, using mixed method approaches to examine household power dynamics and intergenerational influences. Also, relevant policy bodies should implement early empowerment strategies, including mentorship, participatory community programs, and schooling initiatives that equip adolescents with decision-making capacity. It is also important to promote inclusive community engagement that encourages intergenerational decision-making equity and integrate gender-responsive economic strengthening programs.

#### Attitude toward violence

This domain showed the highest national prevalence of high empowerment, increasing from 61.6% in 2008 to 79.2% in 2022. Regional disparities, while significant, have declined over time: Greater Accra and Eastern Region exceeded 90%, whereas northern regions such as Savannah and Upper West remained below 50%. Adolescents and less-educated women consistently lagged, though overall absolute and relative inequalities decreased, suggesting some success in shifting societal norms.

Evidence indicates that education and media exposure play critical roles in fostering rejection of intimate partner violence (UNICEF, 2021). Ghana’s progress may reflect the combined effects of policy interventions, including the Ghana Domestic Violence Act (2007), school-based gender awareness programs, and national advocacy campaigns. The steep decline in the Theil Index (78.7 to 18.2) reveals the narrowing of geographic disparities, suggesting that normative change can diffuse broadly when supported by legislation and public campaigns.

The narrowing wealth gradient (Difference declining from 39.1 to 27.7 percentage points) suggests that attitudinal change has transcended, though not eliminated, economic divides. This aligns with evidence from Africa, demonstrating that comprehensive legal frameworks coupled with community mobilization can accelerate normative shifts (UN Women, 2022). However, persistent pockets of low prevalence in northern regions (e.g., Savannah at 42.6%) show that cultural and religious norms can moderate the diffusion of progressive attitudes.

Therefore, further research should identify which interventions most effectively reduced inequalities and examine the interaction between media exposure, schooling, and cultural norms. Also, key policy institutions should expand violence-prevention curricula, integrate gender awareness into national education programs, and target rural and northern regions where prevalence remains low. Also, community leaders and health workers should be equipped to identify and support women at risk, particularly adolescents and women in under-resourced areas.

#### Geographic, socioeconomic and age-related factors

Across all domains, national improvement coexists with persistent socioeconomic disparities. Social independence remains the slowest to progress, with highly pro-rich and pro-educated concentration. Decision-making shows moderate but variable gains, while attitudes toward violence have improved broadly and more equitably. The decline in Theil Index scores across domains indicates substantial progress in reducing regional inequalities, likely driven by infrastructure expansion, urbanization, media reach, and decentralization of services, particularly in the north (World Bank, 2021). This success stands in contrast to persistent or widening regional disparities observed in other West African nations like Nigeria and Mali, where conflict, ethnic fragmentation, or weaker governance may have hampered geographic equity (Moyo and Dhliwayo, 2019; Langer and Stewart, 2015).

However, this geographic convergence may mask the enduring concentration of gains among higher socio-economic status women, particularly in education and wealth. This is further elaborated on as: while the proportion of the total burden of low empowerment explained by region, education, and wealth (Population Attributable Fraction) has decreased in some domains, the Absolute Concentration Index reveals that socioeconomic status is becoming an even stronger determinant of who benefits from empowerment gains. In essence, inequality is becoming less about *where* a woman lives and more about *who she is* in terms of her education and economic standing. This aligns with global patterns where economic growth and service expansion reduce basic access gaps but often exacerbate inequalities in more complex, asset-based capabilities (Ewerling et al., 2017; World Bank, 2019). The implication is profound: as regional disparities lessen, class-based disparities may become the dominant and most pernicious axis of gender inequality in Ghana.

Furthermore, adolescent girls (15–19) emerged as a consistently and severely disadvantaged group across all domains, constituting a critical life-cycle bottleneck. Their empowerment in social independence remained low (<5% throughout the study period), with a relative inequality ratio of 10.3 in 2022. This points to a possible constellation of restrictive social norms controlling mobility, economic activity, and sexuality, often compounded by early marriage and adolescent pregnancy, particularly prevalent in the northern regions (Ghana Statistical Service, Ghana Health Service, and ICF, 2023). These early constraints can set a trajectory of disempowerment that persists into adulthood, undermining the potential of entire generations (Abera et al., 2020).

Moreover, education emerged as the strongest determinant of empowerment, especially for social independence. The gap between women with higher education (98.4% empowered) and no education (11.3%) is not merely an education gap; it is a proxy for possibly deep, intersecting divides in life opportunities, social networks, economic integration, and exposure to empowering ideas. This suggests that while general education expansion policies are necessary, they may be insufficient on their own. Empowerment-specific interventions must directly address the structural barriers that prevent women with little or no formal schooling from translating basic literacy into tangible autonomy, such as access to credit, secure land rights, affordable childcare, and protection from labour market discrimination (Doss et al., 2015).

Nonetheless, Ghana’s experience both reflects and contrasts with broader evidence. The strong, consistent link between education, wealth, and empowerment is a global constant, evident from Nepal to Nigeria (Guinée, 2014; van Dongen et al., 2024; Makinde et al., 2024; Soetan and Obiyan, 2019). Similarly, the reduction in geographic inequality places Ghana ahead of many sub-Saharan African nations struggling with entrenched regional disparities (Makinde et al., 2024; Abera et al., 2020). However, two aspects stand out as particularly distinctive and warrant further investigation: (1) First, the level of inequality in social independence is exceptionally high and has worsened over time, as indicated by the increasing Absolute Concentration Index (ACI). This suggests that gains in women’s social independence have accrued disproportionately to socioeconomically advantaged groups, while women in poorer, less educated, or rural households have benefited far less. In practical terms, improvements in indicators such as educational attainment and age appear to be increasingly concentrated among women at the upper end of the socioeconomic distribution. The rising ACI therefore signals not only persistent inequality but an intensification of inequity, implying that structural barriers limiting social independence among disadvantaged women remain largely unaddressed. Compared with some comparable settings where expansions in female education and delayed marriage have occurred more evenly across wealth groups (Schuster et al., 2021; Nagashima and Yamauchi, 2023; Hahn et al., 2015), the magnitude and worsening pattern observed here appear unusually pronounced, pointing to context-specific constraints that require further investigation.

(2) Second, the trajectory of inequality in women’s decision-making is characterized by marked fluctuations rather than a steady, linear improvement. Instead of consistent progress, periods of improvement are followed by stagnation or reversal. This non-linear pattern contrasts with findings from other settings, where gradual and sustained gains in women’s household decision-making power have been documented alongside broader social and economic development (Chuta, 2017; Moore et al., 2022; Rettig and Hijmans, 2022). The observed volatility suggests that decision-making autonomy may be particularly sensitive to short-term economic shocks, policy shifts, or changes in household dynamics, such as employment instability or evolving gender norms. Unlike social independence, which is often shaped by long-term investments in education and social structures, decision-making power within households may respond more immediately to contextual changes, resulting in uneven progress over time.

These distinctions may be explained by contextual factors: Ghana’s relatively rapid but uneven economic growth may have disproportionately benefited educated, urban women; the strength of extended family systems may differently mediate women’s agency; and specific national policy mixes may have prioritized attitudinal change and geographic equity over dismantling the structural barriers to economic independence for the poorest women.

### Implication for research, policy and practice

Future research must move beyond documenting disparities to investigating the structural mechanisms that concentrate empowerment gains. Mixed-methods research is essential to unpack how educational advantage translates into social independence, why decision-making empowerment stagnated after 2014, and how restrictive norms are perpetuated across generations in specific subnational contexts. Longitudinal cohort studies tracking girls from adolescence into adulthood are critical to understand life-cycle transitions and identify critical windows for intervention. Methodologically, there is a need to develop and validate context-specific measures of social independence that capture local expressions of autonomy and resource control to better inform program design. Without this foundational research, interventions risk being designed on incomplete evidence.

For policy and practice, the Government of Ghana and the Ministry of Gender, Children and Social Protection should adopt a tiered, domain-specific approach. The most urgent priority is social independence for women with no formal education and those in the poorest wealth quintiles, where the empowerment gap exceeds 85 percentage points. Transformative policies are required, including asset ownership, financial inclusion, labour market access with childcare support, and skills training for low-education women. The second priority targets adolescent girls aged 15–19 years, who have less than 5 % social independence empowerment. Life-cycle programming, school-based empowerment clubs, and mentorship are essential to disrupt intergenerational disempowerment. The third priority is sustained implementation for the decision-making domain through community-embedded norm change programs engaging men, religious leaders, and extended families. The fourth priority is maintaining progress in attitudes toward violence, where national prevalence has reached 79.2 percent, through continued enforcement of the Domestic Violence Act and targeted campaigns in northern regions where acceptance remains above 50 percent.

Across all domains, specific focus must be directed to adolescents, women with no formal education, the poorest wealth quintiles, and northern and rural regions. Programs must monitor equity outcomes using measures such as the Difference and Ratio to ensure benefits reach the most disadvantaged, not merely raise national averages.

Furthermore, scholarship warns that empowerment programmes, while well-intentioned, can inadvertently impose additional responsibilities on women without fundamentally altering underlying gender power relations (Kunz, 2010). Programmes that assume women have available time, resources, and decision-making autonomy to participate may paradoxically reinforce existing inequalities by overlooking structural constraints such as time poverty, unpaid care work, and limited mobility. Future interventions should be designed with explicit attention to these structural barriers and should be evaluated not only for their intended outcomes but also for potential unintended consequences, including increased workload or social backlash.

### Strengths and limitations

This study benefits from a multi-wave nationally representative data spanning two decades, allowing for robust analysis of trends. The application of comprehensive inequality metrics (Difference, Ratio, PAF, PAR, ACI, Theil Index) provides a nuanced, multi-faceted understanding of disparities beyond simple prevalence. However, the limitations include reliance on self-reported data, the potential for social desirability bias, and the aggregate nature of the analysis, which may mask intra-household complexities and more nuanced intersectional experiences. Potential unmeasured cultural determinants may have confounded findings. Also, the cross-sectional nature of the data precludes causality.

Age was dichotomized as 15–19 years (adolescents) versus 20–49 years (adults) due to sample size constraints for detailed age stratification within the HEAT built-in database. This approach may mask heterogeneity among adult women, particularly differences between younger adults (20–29 years), mid-adults (30–39 years), and older adults (40–49 years). Future research should disaggregate the 20–49 age group into meaningful subcategories to better understand life-course dynamics and age-related transitions in empowerment.

The SWPER domains, while validated cross-culturally, use cutoffs derived from Africa-wide analysis. Country-specific recalibration for Ghana has not been formally established, and domain-specific thresholds may differ across countries. Future research should explore the applicability of these cutoffs in the Ghanaian context using qualitative and mixed methods approaches.

Additionally, the SWPER domains, may not fully capture locally relevant expressions of empowerment in Ghana, such as participation in market activities, control over harvest income, or influence in extended family decisions. Future qualitative research should explore these dimensions to complement quantitative findings.

Moreover, the analysis is limited to married/in-union women, excluding never-married, divorced, or widowed women whose empowerment trajectories may differ substantially. This exclusion, required by the SWPER methodology, means our findings do not represent all Ghanaian women.

Nevertheless, the analysis offers a comprehensive framework for monitoring empowerment inequalities and informing targeted interventions.

## Conclusion

Ghana has made notable national gains in women’s empowerment, particularly in shaping equitable attitudes toward violence and narrowing geographic disparities. Yet persistent socioeconomic and age-related inequalities, especially in social independence, highlights the challenge of translating national averages into inclusive empowerment. Targeted, multi-sectoral policies and programs focusing on education, economic inclusion, adolescent empowerment, and structural equity are essential to ensure that all women, regardless of age, wealth, or location, can fully exercise agency and autonomy.

The central challenge for Ghana is no longer raising national averages, but ensuring that progress reaches the poorest, least educated, and youngest women in every region. Without deliberate, equity-focused interventions, the country risks creating a two-track empowerment system where educated, wealthy women advance while millions are left behind. Achieving SDG 5 requires shifting from raising averages to closing gaps, ensuring no woman is left behind.

## Data Availability

All data generated or analyzed during this study are included in this article. The dataset used can be accessed at https://www.who.int/data/inequality-monitor/assessment_toolkit.
